# The frailty phenotype and sarcopenia: Similar but not the same

**DOI:** 10.1002/agm2.12070

**Published:** 2019-06-05

**Authors:** Matteo Cesari

**Affiliations:** ^1^ Department of Clinical Sciences and Community Health University of Milan Milan Italy; ^2^ Geriatric Unit Foundation IRCCS Ca’ Granda Ospedale Maggiore Policlinico Milan Italy

The need for nesting geriatric principles into modern care practice is urgent. Our health‐care systems have been completely underestimating the role played by the aging process in the definition of clinical manifestations and diseases.^1^ Given the fallacy of the standalone disease approach in the evaluation of the older person, several specialties are today looking with a mix of interest and anxiety to the geriatric literature, trying to find here some easy solutions to the aging of their patients and the increasing inadequacy of their protocols. The search inevitably ends at what geriatricians have been saying for years but has largely remained unheard: diseases are not the only center of action at old age, function should be the focus of interventions, the assessment of the individual should be comprehensive, and an integrated and multidisciplinary network of care is crucial. These concepts are difficult to understand, accept, and apply without the necessary background and willingness to change.

In this evolving scenario, words such as *frailty* and *sarcopenia* are increasingly used^2^ and often misused. They are relatively easy to say and “see.” The frailty phenotype is probably the most widely known and adopted measure of frailty.^3^ Its five constituent criteria are easy to remember. Its design as a score also facilitates its implementation as it mirrors existing routine procedures in many settings and disciplines with questionnaires and scales. The five criteria make sense at representing a typical manifestation of the older individual. The characterization of frailty around the physical domain is of immediate acceptance, too; after all, we “see” the fragility of the person in his/her walking, strength, fatigue, weight loss, and inactivity.

On the one hand, the easy implementation of the frailty phenotype has substantially promoted discussions on aging and age‐related conditions beyond the perimeter of geriatric medicine. On the other hand, it has oversimplified some concepts and created false (at least, to me) expectations. It is, today, frequent to perceive how the inner nature of frailty biology is confused with the instrument designed for its measurement. As soon as frailty is translated with the phenotype, it is automatically (and erroneously) forgotten that the age‐related decline of homeostatic reserves^4^ might also be hidden behind an excessive weight gain, a cognitive impairment in a physically fit person, or many signs and symptoms (eg, tremor, vision impairment, hearing loss, and dizziness) other than the five famous criteria.

The simplification of frailty with the phenotype can also be perceived in some automatisms and arguably easy solutions that, as geriatricians, we should reject. For example, the rigid association between the presence of a certain frailty criterion and the proposal of a specific intervention is frequently seen: if the person is losing weight, then introduce nutritional supplements; if the person is sedentary or weak, then recommend physical exercise, and so forth. In reality, we all know that targeting the symptoms is usually the wrong way to go, especially when dealing with the complexity of older persons and geriatric syndromes. If the phenotype is instead accepted for what it is (ie, a clinically evident manifestation of the organism), its results should rather be considered as informing a consequent and coherent cascade of evaluations leading to the identification of the underlying biological causes. Only these latter should be considered reliable targets of interventions. And this is completely in line with the principles of our background in geriatric medicine and evidence on comprehensive geriatric assessment.^5^ Before the frailty phenotype (and/or its components) can be treated as a formal disease, it is necessary to isolate and delineate its specific and unique pathophysiological mechanism (ie, the eventual target for future biology‐driven interventions).

It is also important to open a parenthesis here about another ambiguity in this field: interventions promoting physical activity, healthy diet, social interactions, and so forth should be included in every medical recommendation, independently of the patient's age and physician's specialty. These factors are surely important and, as geriatricians, we well know the magnitude of their benefits in frail patients. At the same time, if we limit the solution of frailty (and/or other geriatric conditions) to the suggestion of lifestyle modifications, we may implicitly demean the role of the entire field of geriatric medicine.

The ambiguities in the use of geriatric terms in non‐geriatric settings are not limited to the *frailty phenotype*. *Sarcopenia* suffers of the same issue, too. For example, an increasing number of studies in the oncology field talks about sarcopenia in cancer patients. *Sarcopenia* is again representing the easy word‐to‐go, but it is not considered that cachexia is more likely to be measured in the presence of a catabolic disease. One word for two different conditions represents a problem.

Sarcopenia can be easily imagined as the main biological foundation of physical frailty, especially if this latter is operationalized following the phenotype model. Nevertheless, it is noteworthy that the Sarcopenia and Physical Frailty in Older People: Multicomponent Treatment Strategies (SPRINT‐T) Consortium decided to exclude the frailty phenotype from the definition of the so‐called physical frailty and sarcopenia condition. The SPRINT‐T is a 48‐million‐euro project funded by the Innovative Medicines Initiatives, currently ongoing across Europe since 2014.^6^ Its aim is to develop the operational definition of a novel nosological condition respecting the requirements asked by regulatory agencies and paving the way for future pharmacological interventions against skeletal muscle decline. The definition designed by the SPRINT‐T Consortium has been preliminarily endorsed by the European Medicines Agency. It translates the concept of physical frailty with the impairment captured by the Short Physical Performance Battery (SPPB), in the absence of mobility disability.^7^ Why did the SPRINT‐T project choose the SPPB instead of the frailty phenotype for capturing the clinical manifestation of the condition of interest? The SPPB is a robust, validated, replicable measure of physical performance based on timed tests. It does not rely on questionnaires or self‐reported symptoms that might bias the objective evaluation of the underlying muscle biology. In contrast to the frailty phenotype, the SPPB indeed represents a direct assessment of the muscle in action. In 2019, the SPRINT‐T trial^8^ will end. If successful, it will show that a muscle‐centered condition of physical frailty (ie, abnormal SPPB) may represent an ideal target for applying (pharmacological and non‐pharmacological) preventive interventions against disability in older persons. It is noteworthy that, according to preliminary findings,^9^ the SPRINT‐T population is frankly overweight (ie, 28.6 kg/m^2^), which contradicts the idea that frailty can only be found in lean persons. This is not particularly surprising considering the large body of evidence showing the active role played by adipose tissue in the deterioration of the skeletal muscle and the increasing frailty of an individual.^10^

In conclusion, I believe that the frailty phenotype finds in sarcopenia a substantial, but not exhaustive pathogenetic explanation. To date, thanks to its diffusion, the frailty phenotype might be used to inform non‐geriatric disciplines about the existence of unmet clinical needs and recommend more attention to neglected signs, symptoms, and conditions of old age. Among these, age‐related skeletal muscle decline is surely one of the most important. Nevertheless, the phenotype should not be overestimated in its properties and capacities. It remains (at least, to date) an instrument able to identify a specific population with a quite heterogeneous biological background, sometimes due to sarcopenia, many other times probably not.

## CONFLICTS OF INTEREST

Dr. Cesari acts as Workpackage Leader of the SPRINT‐T Consortium, which, through the Innovative Medicines Initiatives, is partly funded by the European Federation of Pharmaceutical Industries and Associations (EFPIA).

## References

[1] Tinetti M , Fried T . The end of the disease era. Am J Med. 2004;116:179‐185. 14749162 10.1016/j.amjmed.2003.09.031

[2] Woo J . Combating frailty and sarcopenia in aging populations: switching to a more positive paradigm. Aging Med. 2019;2(1):7‐10. 10.1002/agm2.12052 PMC6880689 31942507

[3] Fried L , Tangen C , Walston J , et al. Frailty in older adults: evidence for a phenotype. J Gerontol A Biol Sci Med Sci. 2001;56:M146‐M156. 11253156 10.1093/gerona/56.3.m146

[4] Morley J , Vellas B , Abellan van Kan G , et al. Frailty consensus: a call to action. J Am Med Dir Assoc. 2013;14:392‐397. 23764209 10.1016/j.jamda.2013.03.022 PMC4084863

[5] Chen X , Yan J , Wang J , Yu P , Geriatric, Gerontology Branch of Chinese Medicine Association . Chinese expert consensus on the application of comprehensive geriatric assessment for the elderly. Aging Med. 2018;1:100‐105. 10.1002/agm2.12019 PMC6880693 31942485

[6] Bernabei R , Mariotti L , Bordes P , Roubenoff R . The ‘Sarcopenia and Physical fRailty IN older people: multi‐componenT Treatment strategies’ (SPRINTT) project: advancing the care of physically frail and sarcopenic older people. Aging Clin Exp Res. 2017;29:1‐2. 10.1007/s40520-016-0707-2 28144913

[7] Cesari M , Landi F , Calvani R , et al. Rationale for a preliminary operational definition of physical frailty and sarcopenia in the SPRINTT trial. Aging Clin Exp Res. 2017;29:81‐88. 28188558 10.1007/s40520-016-0716-1

[8] Landi F , Cesari M , Calvani R , et al. The ‘Sarcopenia and Physical fRailty IN older people: multi‐componenT Treatment strategies’ (SPRINTT) randomized controlled trial: design and methods. Aging Clin Exp Res. 2017;29:89‐100. 28144914 10.1007/s40520-016-0715-2

[9] Marzetti E , Cesari M , Calvani R , et al. The ‘Sarcopenia and Physical fRailty IN older people: multi‐componenT Treatment strategies’ (SPRINTT) randomized controlled trial: case finding, screening and characteristics of eligible participants. Exp Gerontol. 2018;113:48‐57. 30261246 10.1016/j.exger.2018.09.017

[10] Delmonico M , Harris T , Visser M , et al. Longitudinal study of muscle strength, quality, and adipose tissue infiltration. Am J Clin Nutr. 2009;90:1579‐1585. 19864405 10.3945/ajcn.2009.28047 PMC2777469

